# Host-pathogen relationship in retreated tuberculosis with major rifampicin resistance–conferring mutations

**DOI:** 10.3389/fmicb.2023.1187390

**Published:** 2023-07-04

**Authors:** Nguyen Thi Le Hang, Minako Hijikata, Shinji Maeda, Pham Huu Thuong, Hoang Van Huan, Nguyen Phuong Hoang, Do Bang Tam, Pham Thu Anh, Nguyen Thu Huyen, Vu Cao Cuong, Nobuyuki Kobayashi, Keiko Wakabayashi, Akiko Miyabayashi, Shintaro Seto, Naoto Keicho

**Affiliations:** ^1^NCGM-BMH Medical Collaboration Center, Hanoi, Vietnam; ^2^Department of Pathophysiology and Host Defense, The Research Institute of Tuberculosis, JATA, Tokyo, Japan; ^3^Faculty of Pharmaceutical Sciences, Hokkaido University of Science, Sapporo, Hokkaido, Japan; ^4^My Duc District General Hospital, Hanoi, Vietnam; ^5^Hanoi Lung Hospital, Hanoi, Vietnam; ^6^Department of Microbiology, Hanoi Lung Hospital, Hanoi, Vietnam; ^7^Department of Biochemistry, Hematology and Blood Transfusion, Hanoi Lung Hospital, Hanoi, Vietnam; ^8^Tuberculosis Network Management Office, Hanoi Lung Hospital, Hanoi, Vietnam; ^9^Department of Health Policy and Economics, Hanoi University of Public Health, Hanoi, Vietnam; ^10^Hanoi Department of Health, Hanoi, Vietnam; ^11^Department of Internal Medicine, Fureai Machida Hospital, Tokyo, Japan; ^12^The Research Institute of Tuberculosis, JATA, Tokyo, Japan; ^13^National Center for Global Health and Medicine, Tokyo, Japan

**Keywords:** *Mycobacterium tuberculosis*, recurrence, rifampicin resistance, rpoB variants, whole genome sequencing, blood transcriptomic signatures, interferon-inducible genes

## Abstract

**Introduction:**

It is assumed that host defense systems eliminating the pathogen and regulating tissue damage make a strong impact on the outcome of tuberculosis (TB) disease and that these processes are affected by rifampicin (RIF) resistance–conferring mutations of *Mycobacterium tuberculosis* (Mtb). However, the host responses to the pathogen harboring different mutations have not been studied comprehensively in clinical settings. We analyzed clinico-epidemiological factors and blood transcriptomic signatures associated with major *rpoB* mutations conferring RIF resistance in a cohort study.

**Methods:**

Demographic data were collected from 295 active pulmonary TB patients with treatment history in Hanoi, Vietnam. When recruited, drug resistance–conferring mutations and lineage-specific variations were identified using whole-genome sequencing of clinical Mtb isolates. Before starting retreatment, total RNA was extracted from the whole blood of HIV-negative patients infected with Mtb that carried either the *rpoB* H445Y or *rpoB* S450L mutation, and the total RNA was subjected to RNA sequencing after age-gender matching. The individual RNA expression levels in the blood sample set were also measured using real-time RT-PCR. Logistic and linear regression models were used to assess possible associations.

**Results:**

In our cohort, *rpoB* S450L and *rpoB* H445Y were major RIF resistance–conferring mutations [32/87 (36.8%) and 15/87 (17.2%), respectively]. H445Y was enriched in the ancient Beijing genotype and was associated with nonsynonymous mutations of Rv1830 that has been reported to regulate antibiotic resilience. H445Y was also more frequently observed in genetically clustered strains and in samples from patients who had received more than one TB treatment episode. According to the RNA sequencing, gene sets involved in the interferon-γ and-α pathways were downregulated in H445Y compared with S450L. The qRT-PCR analysis also confirmed the low expression levels of interferon-inducible genes, including *BATF2* and *SERPING1*, in the H445Y group, particularly in patients with extensive lesions on chest X-ray.

**Discussion:**

Our study results showed that *rpoB* mutations as well as Mtb sublineage with additional genetic variants may have significant effects on host response. These findings strengthen the rationale for investigation of host-pathogen interactions to develop countermeasures against epidemics of drug-resistant TB.

## Introduction

Management of tuberculosis (TB) has been challenged by increased resistance to anti-TB drugs, especially rifampicin (RIF), which has long been the backbone of the standard short-course chemotherapy against *Mycobacterium tuberculosis* (Mtb). In 2021, the estimated proportion of people with TB who had RIF-resistant TB with or without isoniazid-resistance was 3.6% among new cases and 18% among those previously treated, which continues to be public health threat.^1^ Globally, it is estimated that transmission of multi-drug resistant (MDR) TB strains is responsible for over 70% of MDR-TB cases (Kendall et al., 2015).

RIF binds to the beta-subunit of RNA polymerase (RpoB) and kills bacteria by inhibiting their critical RNA synthesis (Chauhan et al., 2021). In Mtb, a variety of RIF resistance–conferring mutations are located in the 81-bp RIF resistance–determining region (RRDR) of the *rpoB* gene, corresponding to codons 426–452 of the beta-subunit. Within the RRDR, amino acid substitutions at codons 450 and 445 are enriched and are associated with high-level resistance to RIF (Unissa and Hanna, 2017).

Vietnam is one of the 30 countries with high TB burden, with an estimated incidence rate of 173 (112–247) per 100,000 population, and also one of the 30 countries with high MDR/RIF-resistance TB burden, with 8,900 patients estimated to have MDR-TB in 2021 (see footnote 1). In Vietnam, the most prevalent RIF-resistant mutations were found at codon 450 (37.8%), followed by codon 445 (23.0%) in the RRDR of the *rpoB* gene of 74 strains (Minh et al., 2012). Regarding the genetic background of Mtb in Vietnam, Beijing strains belonging to lineage 2 of Mtb are mainly spreading in the urban areas (Maeda et al., 2014).

The *rpoB* mutations affect bacterial biological processes via the functional modulation of RNA polymerase differently, and thus may regulate the host response. The *rpoB* H445Y mutation in Mtb is associated with an increased transcription elongation rate (Stefan et al., 2018), which is also the case for equivalent *Escherichia coli* mutations (Rasouly et al., 2021). In the earlier reports, a panel of clinically common *rpoB* mutations has been reported to upregulate the phthiocerol dimycocerosate biosynthetic pathway (Bisson et al., 2012) and to change the Mtb cell wall lipid composition (Lahiri et al., 2016).

According to a recent report (Howard et al., 2018), macrophage activation occurs via aerobic glycolysis when infected with Mtb strains harboring *rpoB* S450L mutation in mice, whereas H445Y-carrying strains preferentially induce interferon (IFN)-β, driving less effective aerobic glycolysis, presumably through the mutation-mediated modulation of mycobacterial lipid components. It is widely accepted that during the early phase of Mtb infection, the pathogen provokes IL-1β production and macrophage glycolytic reprogramming with decreased oxidative phosphorylation that act toward bacterial elimination (Collins et al., 2021; Silvério et al., 2021). However, Mtb escapes into cytosol using the mycobacterial ESX-1 system, induces IFN-β production and inhibits aerobic glycolysis with IL-1β induction (Howard and Khader, 2020; Olson et al., 2021). If the bacteria survive and persist by migrating into the lung interstitial tissue that leads to the granuloma formation, maintenance of aerobic glycolysis in surrounding macrophages may rather contribute to immune pathology with detrimental effects (Sheedy and Divangahi, 2021). It is thus assumed that host defense systems eliminating the pathogen and regulating tissue damage make a strong impact on the outcome of TB disease and that these processes are affected by different RIF mutations in clinical settings. In this context, it is imperative to investigate the overall effects of drug-resistant Mtb strains in TB patients (Allué-Guardia et al., 2021).

For this approach, we should also consider that the effects of drug resistance mutations are complicated by their secondary genomic change, including well-known compensatory mutations (Alifano et al., 2015; Stefan et al., 2018) and other mutations that regulate the early resumption of bacterial growth after drug exposure. The latter ones have recently been reported as “antibiotic resilience” mutations, and associated with poor treatment outcome (Liu et al., 2022).

In addition, gene expression signatures induced by type I and/or II IFNs are considered as blood-based biomarkers of host response in TB. Among these, basic leucine zipper ATF-like transcription factor 2 (*BATF2*) is frequently identified as a key gene (Singhania et al., 2018). BATF2 induces inflammatory responses in lung recruited macrophages, which in turn leads to deleterious inflammation and contributes to TB disease progression in a murine model (Guler et al., 2019).

In this study, we thus hypothesized that Mtb strains with different *rpoB* mutations may result in different host responses, with the possible involvement of Mtb genetic factors or IFN signaling pathway. We described the characteristics of the RIF resistance–conferring mutations observed in sputum smear-positive active TB patients who had previously received TB treatment in Hanoi, Vietnam, and assessed the relationship between clinico-epidemiological and pathogen factors. We further investigated transcriptomic signatures in peripheral blood obtained from patients infected with Mtb harboring major *rpoB* mutations, H445Y or S450L, using differentially expressed genes (DEG) analysis for significantly up-or down-regulated genes and gene set enrichment analysis (GSEA) to characterize differences in genome-wide expression profiles between the groups (Subramanian et al., 2005), and discussed the clinical relevance of these genomic variations of Mtb to the host response.

## Materials and methods

### Ethics statement

This study was approved by the Ethical Committees of the Hanoi Department of Health, Vietnam, the National Center for Global Health and Medicine, and the Research Institute of Tuberculosis, Japan Anti-Tuberculosis Association, Japan (RITIRB29-35). Written informed consent was obtained from all patients.

### Study sites, patient recruitment, and sample collection

In our prospective study on recurrent TB, we included 11 of 12 urban districts in Hanoi city and Hanoi Lung Hospital, a referral hospital for all TB patients in Hanoi, as the catchment area, where more than 60% of TB patients in the city were diagnosed and treated during the study period. Patients were considered eligible if they were aged 18 years or older, resided in the catchment area, self-declared that they had suffered from TB accompanied with TB treatment courses, currently diagnosed with smear-positive pulmonary TB, and agreed to participate in this study. Eligible patients who visited the local TB care units and Hanoi Lung Hospital were recruited consecutively from December 2012 to November 2015.

Information about previous TB treatment, including completion of the standard regimen and registration to the National TB Program in Hanoi city, was based on interviews conducted by pre-trained healthcare staff. Before initiating anti-TB treatment, sputum specimens were cultured and subjected to identification of Mtb, DNA extraction for molecular typing, and whole-genome sequencing. The bacterial load in sputum smear and affected lesions on chest X-ray were assessed to estimate the severity of the disease.

### Whole-genome sequencing of *Mycobacterium tuberculosis* DNA samples

DNA samples were extracted from Mtb strains using an Isoplant kit (Nippon Gene, Tokyo, Japan) and analyzed using Illumina HiSeq and MiSeq systems (Illumina, San Diego, CA, United States). For HiSeq 2500, libraries were prepared using a TruSeq DNA PCR Free Sample Prep Kit (Illumina), and paired-end (2 × 150 bp) sequencing was performed. For Miseq, libraries were prepared with a QIAseq FX DNA Library Kit (QIAGEN GmbH, Hilden, Germany), and paired-end (2 × 250 bp) sequencing was used.

Sequence reads were trimmed and mapped to the H37Rv genome (AL123456.3) using BWA-MEM 0.7.15.^2^ After excluding severely contaminated samples, variant calling was performed with a Genome Analysis Toolkit (GATK version 3.7).^3^ Drug resistance–conferring mutations, including small indels and lineage-specific variations, and major sublineages were identified using TB-Profiler version 4.3.^4^ Ancient and modern Beijing sublineages were indicated by the absence and presence of the C1477596T (*ogt*12) mutation, a surrogate for a copy of IS*6110* in the NTF region (Mestre et al., 2011), and further classified using a recently proposed SNV-based genotyping scheme for lineage 2 strains (Thawornwattana et al., 2021). Pairwise single-nucleotide variation (SNV) differences were calculated using a list of concatenated variants with an in-house Python script described in a previous study (Maeda et al., 2020).

### Host RNA sequencing and downwards analyses

A volume of 2.5 mL of peripheral blood was collected from each patient into a PAXgene blood RNA tube (PreAnalytiX, Hombrechtikon, Switzerland) before starting retreatment, and total RNA was extracted using a PAXgene Blood RNA Kit (QIAGEN). RNA sequencing was performed for six HIV-negative age-gender matched pairs of patients infected with Mtb strains that harbored either the *rpoB* S450L or *rpoB* H445Y mutation. Libraries were prepared with TruSeq Stranded mRNA Library Prep (Illumina), and a QIAseq FastSelect Globin Kit (QIAGEN) was combined to avoid generations of reads from globin mRNAs. Sequencing (2 × 75 bp) was performed in NextSeq500 (Illumina). Pair-end reads were aligned to human reference genome GRCh38.primary_assembly.genome.fa with gencode.v38.primary_assembly.annotation.gtf^5^ using STAR aligner v2.7.9a.^6^ Mapped reads associated with each gene were counted using featureCounts v2.0.1 (Liao et al., 2014).

#### Principal component and hierarchical clustering analysis

To identify possible groups within our dataset of patients’ blood mRNA signatures, hierarchical clustering on principal components was performed with the Ward’s criterion on the selected principal components and K-means for further clustering to improve the initial partition obtained from hierarchical clustering using the FactoMineR v2.4 and factoextra v1.0.7 packages (Lê et al., 2008).

#### Estimation of blood cell types and their characteristics

CIBERSORTx^7^ (Newman et al., 2019) was used to estimate the proportion of blood cell types from RNA-seq data using LM22, a signature matrix for profiling 22 functionally defined human immune cell types, in the above website. Cell marker enrichment analysis was also performed using clusterProfiler v4.2.0 (Wu et al., 2021) with the CellMarker database (Zhang et al., 2019). Blood cell differentials directly obtained from complete blood count measurements were also compared with the above estimates.

#### Extraction of differentially expressed genes from RNA-seq data

DEGs were analyzed using an R package, DESeq2 v1.26.0 (Love et al., 2014) with adjusted *p* values of <0.1 set as the default. Comparison of host mRNA expression in peripheral blood between patient groups infected with Mtb harboring *rpoB* S450L and *rpoB* H445Y was made using generalized linear models, assuming a negative binomial distribution in DESeq2, which enabled us to analyze pairs of samples before and after controlling for major clusters generated from gene expression patterns using the hierarchical clustering on principal components analysis method. Gene expression levels were visualized with heatmaps drawn with the pheatmap v1.0.12 or heatmap.2 packages in gplots v3.1.1.1.^8^

#### Functional enrichment analysis

To further determine the characteristics of each cluster or of a gene set possibly showing differential expression between H445Y and S450L groups, functional enrichment analysis was performed using up-to-date annotation systems, Gene Ontology (GO) and the Kyoto Encyclopedia of Genes and Genomes. Significant GO terms and signal pathways were screened when the *p* value was <0.05.

#### Gene Set enrichment analysis

GSEA was performed using an R function in clusterProfiler v4.2.0 (Wu et al., 2021). Expression datasets by whole genes were ranked using a metric: −log10(p value)*sign(log2FC). The pre-ranked gene list and phenotype information (*rpoB* S450L or *rpoB* H445Y) were uploaded to GSEA for enrichment analysis with default parameters. The pre-defined hallmark gene sets in the Molecular Signatures Database (MSigDB, Broad Institute, Cambridge, MA, United States) were selected for analysis (Subramanian et al., 2005). The false discovery rate was used to correct for multiple comparisons (Benjamini et al., 2001).

#### Measurement of RNA expression levels of functional genes by real-time RT-PCR

Total RNA was reverse-transcribed with SuperScript III reverse transcriptase (Thermo Fisher Scientific, Waltham, MA, United States) with random nonamers (TaKaRa, Shiga, Japan), and cDNA was subjected to qPCR using TaqMan® GeneExpression Assays (Thermo Fisher; Supplementary Table S1) using StepOne Plus (Thermo Fisher). GAPDH was used to normalize the values of the target gene expression using the ΔΔCt method, and the relative expression level of each gene was calculated to a fixed control complementary DNA throughout the study.

### Statistical analyses

Chi-squared or Fisher’s exact test was performed to compare the frequencies of events, and Wilcoxon’s rank-sum test was used to compare non-parametric distributions between the groups. Possible associations between Mtb *rpoB* mutations and other factors were studied using logistic and linear regression models. Changes in host gene expression following Mtb genetic variants were studied using univariate and multivariate linear regression models. The analyzes were performed using STATA version 16 (StataCorp LLC, College Station, TX, United States). All factors used in the univariate analysis, including Mtb sublineages, were added to the multivariate model for adjustment due to their biological importance. *p* values less than 0.05 were considered statistically significant.

## Results

### Characteristics of the study population

In total, 295 adult patients with one or more episodes of treatment for active pulmonary TB were recruited. Their median age was 46.8 years (interquartile range IQR 37.1–54.8), and 87.8% were male. HIV-positive patients accounted for 3.1% (9/295) of the cohort. Of the recruited patients, 83.7% (247/295) had received a single treatment episode, and 16.3% (48/295) had received more than one episode of treatment in the past. The median interval between the initial and current episode was 3 years (IQR 1–6), and treatment discontinuation during the initial episode was observed in 14.2% (42/295) of patients due to individual reasons, including patient’s self-discontinuation or drug adverse effects (Supplementary Table S2).

### Distribution of rifampicin resistance–conferring mutations in the study area

After excluding the cases in which sputum cultures were negative, contaminated, or unavailable, clinical isolates of Mtb from 232 patients were subjected to DNA extraction. Of these, 215 samples with sufficient DNA quality were further analyzed for identification of drug resistance–conferring mutations using whole-genome sequencing. Eighty-seven isolates had RIF resistance–conferring mutations listed in the WHO catalog,^9^ which were all identified within *rpoB*; 98.9% (86/87) were located within RRDR, and 95.4% (83/87) were MDR-TB accompanied by a major isoniazid resistance–conferring mutation, *kat*G S315T (80/83 or 96.4%) (data not shown). Of these 87 *rpoB* mutations, S450L was the most frequent [36.8% (32/87)], and mutations at the H445 position ranked next [31.0% (27/87)], of which H445Y was predominant [17.2% (15/87)], and other *rpoB* mutations included those at the D435 position [9.2% (8/87)] (Table 1).

**Table 1:** Known mutations identified in *rpoB* (A); and distribution of mutations among Mtb lineage/sublineage groups (B).

(A) Category	Frequency (*N*)	% to the strains analyzed (*N* = 215)	% to the strains harboring *rpoB* mutations (*N* = 87)
**No mutation**	**128**	**59.5**	
**With mutation**	**87**	**40.5**	100.0
***rpoB*** **S450L**	**32**	**14.9**	**36.8**
Only S450L	26	12.1	29.9
Co-existance with other mutations (D435V, R552C, S441L, L527V, G332R)	6	2.8	6.9
***rpoB*** **H445Y**	**15**	**7.0**	**17.2**
Only H445Y	9	4.2	10.3
Co-existance with other mutations (S428R, Q429H, S441L, L452P, D435V, S450L)	6	2.8	6.9
**Other mutations**	**40**	**18.6**	**46.0**
*rpoB* D435F	1	0.5	1.2
*rpoB* D435V	3	1.4	3.5
*rpoB* D435Y	4	1.8	4.7
*rpoB* H445D	9	4.2	10.3
*rpoB* H445G	1	0.5	1.2
*rpoB* H445N	1	0.5	1.2
*rpoB* H445R, *rpoB* Q429H	1	0.5	1.2
*rpoB* I491F	1	0.5	1.2
*rpoB* L430P	2	0.9	2.3
*rpoB* L430P, *rpoB* D435G	1	0.5	1.2
*rpoB* L430P, *rpoB* N437S	1	0.5	1.2
*rpoB* L452P	7	3.2	8.1
*rpoB* Q432E, or *rpoB* Q432L, or *rpoB* Q432P	3	1.4	3.5
*rpoB* S441L	4	1.8	4.6
*rpoB* S450F, *rpoB* L449M	1	0.5	1.2

(B) Mtb lineage/sublineage	*rpoB* mutations, presented in frequency and proportion to each Mtb lineage/sublineage			
	No mutation	*rpoB* H445Y	*rpoB* S450L	Other *rpoB* mutations
Non-Beijing (*N* = 38)	25 (65.8)	1 (2.6)	10 (26.3)	2 (5.3)
Ancient Beijing (*N* = 124)	75 (60.5)	11 (8.9)	11 (8.9)	27 (21.8)
Modern Beijing (*N* = 53)	28 (52.8)	3 (5.7)	11 (20.8)	11 (20.8)
Total (*N* = 215)	128 (59.5)	15 (6.9)	32 (14.9)	40 (18.6)
*P* value*	0.008			
*P* value**	0.018			

*By Fisher’s exact test, between three groups (*rpoB* S450L, *rpoB* H445Y, and other mutations). **By Fisher’s exact test, between four groups (no mutation, *rpoB* S450L, *rpoB* H445Y, and other mutations). Mtb, *Mycobacterium tuberculosis*.

### Genomic features associated with RIF resistance–conferring mutations

#### *rpoB* mutations and *Mycobacterium tuberculosis* lineages/sublineages

Beijing genotype strains accounted for 82.3% (177/215) of the strains isolated in our study, and these strains were divided into modern and ancient Beijing genotypes, 24.7% [53/215] and 57.7% [124/215] respectively, of which major subgroups were L2.2.M.6.1 [47.2% (25/53)] and L2.2.AA.3.1. [72.6% (90/124)] respectively (Thawornwattana et al., 2021). One isolate belonged to proto-Beijing (lineage 2.1) group. Other non-Beijing strains consisted of lineages 1 and 4, 4.2% [9/215] and 13.0% [28/215], respectively (data not shown). *rpoB* mutations were present in 41.8% (74/177) of the Beijing strains. Modern Beijing and non-Beijing strains harbored *rpoB* H445Y (5.7 and 2.6%, respectively) less frequently than S450L (20.8 and 26.3%, respectively), whereas in ancient Beijing strains the H445Y and S450L mutations were carried equally (8.9 and 8.9%). The distribution of these mutations was thus significantly different depending on the lineage/sublineage (*p* = 0.018; Table 1). Logistic regression models showed that H445Y was positively associated with the ancient Beijing genotype in univariate (odds ratio OR = 10.00 [95% confidence interval CI 1.09–91.98]) and multivariate analysis after adjustment for Mtb genotypic clusters, compensatory mutations and patients’ age, gender and body mass index (BMI) [adjusted OR = 20.67 (1.04–410.95)] (Table 2), as compared with S450L.

**Table 2:** Pathogen and host factors associated with *rpoB* H445Y vs. *rpoB* S450L mutations in univariate or multivariate analysis using logistic models.

Pathogen and host factors	*rpoB* H445Y vs. *rpoB* S450L			
	OR	95% CI	aOR	95% CI
Ancient Beijing vs. non-Beijing genotype	**10.00**	**1.09–91.98**	**20.67**	**1.04–410.95**
Modern Beijing vs. non-Beijing genotype	2.73	0.24–30.66	3.66	0.17–77.95
Compensatory mutation*	**0.07**	**0.02–0.32**	**0.08**	**0.01–0.61**
Genotypic clustered**	3.52	0.68–18.30	9.41	0.37–236.22
Age (increased by 1 year)	0.97	0.92–1.02	0.96	0.90–1.04
Gender (female vs. male)	1.79	0.46–6.97	2.22	0.25–19.60
Body mass index (increased by one unit)	0.77	0.56–1.05	0.74	0.47–1.16

*Any mutation in *rpoA, rpoC*, or non-RRDR *rpoB* gene. **Defined by <6 pairwise SNV difference. RRDR, Rifampicin resistance–determining region; SNV, single-nucleotide variation; OR, odds ratio; aOR, adjusted odds ratio; CI, confidence interval. Values in bold are statistically significant.

#### *rpoB* mutations and their compensatory mutations

Whereas 87.5% (28/32) of *Mtb* strains harboring S450L carried at least one nonsynonymous mutation in the *rpoA*, *rpoC*, or non-RRDR *rpoB* gene, which is considered to be a possible compensatory mutation (Merker et al., 2018), only 33.3% (5/15) of H445Y strains had them (*p* < 0.001; Supplementary Table S3). The logistic regression models also demonstrated that H445Y was inversely associated with potentially compensatory mutations in univariate and multivariate analysis [OR = 0.07 (0.02–0.32), adjusted OR = 0.08 (0.01–0.61), respectively; Table 2]. The frequencies of coexistent *katG* S315T, the most prevalent isoniazid resistance–conferring mutation, were not different between H445Y [93.3% (14/15)] and S450L [87.5% (28/32)] (*p* > 0.999, data not shown).

#### *rpoB* mutations and Rv1830 mutations potentially regulating “antibiotic resilience”

Three missense variants (R66H, S100F, and V113A) in the mutation hotspots of the Rv1830 gene that has recently been reported to regulate “antibiotic resilience” (Liu et al., 2022) and another variant at the nucleotide position 2,075,516, which disrupts the stop codon of this gene, were found (Supplementary Table S4A). The distributions of these four variants were significantly different among Mtb strains carrying *rpoB* H445Y (20.0%, 3/15), *rpoB* S450L (3.1%, 1/32), other *rpoB* mutations (2.5%, 1/40), or non-*rpoB* mutations (0.8%, 1/128, *p* = 0.007; Supplementary Table S4B). By multivariate analysis, the presence of these Rv1830 mutations was associated with H445Y (adjusted OR = 32.14, 95% CI 1.10–939.06; Supplementary Table S4C), after adjustment for potential confounding factors including Mtb lineage/sublineage and genotypic clusters. In addition, their proportions were also higher in MDR compared with non-MDR [6.0% (5/83) vs. 0.8% (1/132), *p* = 0.033] and in patients with a history of multiple TB treatments compared with a single treatment [12.5% (4/32) vs. 1.1% (2/183), *p* = 0.005; Supplementary Table S4B].

### Epidemiological findings associated with different *rpoB* mutations

#### *rpoB* mutations and *Mycobacterium tuberculosis* genotypic clusters

The proportions of genotypic clusters defined by <6 pairwise SNV differences were different depending on the types of *rpoB* mutations (*p* = 0.020; Supplementary Table S5). When using logistic regression models, H445Y was significantly associated with clustered strains defined by <6 SNVs in both univariate and multivariate analysis after adjustment for patient age, gender, BMI, and Mtb lineages/sublineages [OR = 7.39 (1.81–30.21) and adjusted OR = 11.53 (2.39–55.65)], whereas S450L and other *rpoB* mutations were not (Table 3). The association of H445Y remained significant, and ancient Beijing strains were also associated with the clusters when defined by <12 SNVs (Table 3).

**Table 3:** Pathogen and host factors including *rpoB* mutations possibly associated with Mtb genotypic clusters defined by <6 (A) or < 12 (B) pairwise SNV differences in univariate and multivariate analysis using logistic regression models.

Pathogen and host factors	(A) genotypic clusters (<6 SNVs)				(B) genotypic clusters (<12 SNVs)			
	OR	95% CI	aOR	95% CI	OR	95% CI	aOR	95% CI
Ancient Beijing vs. non-Beijing genotype	1.14	0.30–4.30	0.78	0.17–3.52	**4.41**	**1.27–15.28**	**4.50**	**1.23–16.45**
Modern Beijing vs. non-Beijing genotype	0.95	0.20–4.53	0.63	0.12–3.03	3.06	0.79–11.82	2.80	0.70–11.17
Age (increased by 1 year)	0.98	0.94–1.02	0.97	0.93–1.02	0.99	0.97–1.02	0.99	0.97–1.03
Gender (female vs. male)	0.86	0.19–3.97	0.42	0.08–2.31	0.99	0.38–2.62	0.80	0.28–2.30
Body mass index (increased by one unit)	1.16	0.97–1.37	1.24	1.03–1.49	1.05	0.93–1.18	1.06	0.93–1.21
*rpoB* mutations*								
H445Y	**7.39**	**1.81–30.21**	**11.53**	**2.39–55.65**	**3.40**	**1.09–10.56**	**3.32**	**1.01–10.91**
S450L	2.10	0.50–8.91	2.11	0.46–9.69	1.99	0.81–4.91	2.61	0.99–6.84
Other	2.90	0.84–10.09	3.07	0.84–11.23	2.18	0.96–4.97	1.95	0.84–4.50

*No mutation was set as the reference category. Mtb, *Mycobacterium tuberculosis*; SNV, single-nucleotide variation; OR, odds ratio; aOR, adjusted odds ratio; CI, confidence interval. Values in bold are statistically significant.

#### *rpoB* mutations and host background, including episodes of previous tuberculosis treatment

We further investigated patient characteristics associated with the two major *rpoB* mutations, S450L and H445Y. The proportions of patients with multiple episodes of anti-TB treatment were significantly different between these groups; 46.7% (7/15) for H445Y, 21.9% (7/32) for S450L, 12.5% (5/40) for others, and 10.2% (13/128) for wild-type *rpoB* (*p* = 0.001; Supplementary Table S6). The proportion of patients with a BMI <17.0, indicating moderate or severe underweight, were also significantly different between these groups: 60.0% (9/15) for *rpoB* H445Y, 18.8% (6/32) for S450L, 35.0% (14/40) for others, and 32.0% (41/128) for wild-type (*p* = 0.045), whereas there were no significant differences in patients’ age, history of BCG vaccination, clinical manifestations assessed by chest X-ray, sputum smear and culture grades, HIV status, and the interval between the initial and current TB episodes between these groups (Supplementary Table S6). When using logistic regression models, H445Y, but not S450L, was associated with multiple episodes of TB treatment, either by univariate analysis (OR = 6.13, 95% CI 1.54–24.37) or multivariate analysis (adjusted OR = 6.74, 95% CI 1.53–29.72) after adjustment for patients’ age, gender, BMI, Mtb lineage/sublineage, or completion of the initial treatment, when “other *rpoB* mutations” was set as a reference group (Table 4). Similar results were obtained when “no *rpoB* mutations” was set as a reference group (Table 4).

**Table 4:** Factors possibly associated with multiple episodes of tuberculosis treatment in univariate and multivariate analysis using logistic models.

(A) Pathogen and host factors	OR	95% CI	aOR	95% CI
Age (increased by 1 year)	0.99	0.96–1.03	1.01	0.97–1.06
Gender (female vs. male)	0.99	0.32–3.09	1.04	0.24–4.45
Body mass index (increased by one unit)	**0.83**	**0.70–0.98**	0.80	0.62–1.04
Complete vs. incomplete initial treatment	**2.99**	**1.22–7.32**	1.66	0.37–7.34
Ancient Beijing vs. non-Beijing genotype	1.54	0.49–4.84	1.27	0.19–8.32
Modern Beijing vs. non-Beijing genotype	1.74	0.49–6.13	2.51	0.39–16.40
H445Y vs. other *rpo*B mutations	**6.13**	**1.54–24.37**	**6.74**	**1.53–29.72**
S450L vs. other *rpo*B mutations	1.96	0.56–6.89	2.07	0.51–8.37

(B) Pathogen and host factors	OR	95% CI	aOR	95% CI
Age (increased by 1 year)	0.99	0.96–1.03	1.01	0.97–1.04
Gender (female vs. male)	0.99	0.32–3.09	0.86	0.23–3.13
Body mass index (increased by one unit)	**0.83**	**0.70–0.98**	0.84	0.70–1.01
Complete vs. incomplete initial treatment	**2.99**	**1.22–7.32**	**3.02**	**1.11–8.18**
Ancient Beijing vs. non-Beijing genotype	1.54	0.49–4.84	1.51	0.43–5.25
Modern Beijing vs. non-Beijing genotype	1.74	0.49–6.13	1.79	0.47–6.81
H445Y vs. no *rpo*B mutation	**7.74**	**2.41–24.82**	**9.53**	**2.63–34.57**
S450L vs. no *rpo*B mutation	2.48	0.90–6.84	2.80	0.93–8.40
Other vs. no *rpo*B mutation	1.26	0.42–3.79	1.29	0.40–4.07

(A): H445Y/S450L compared with other *rpoB* mutations, (B): H445Y/S450L compared with no *rpoB* mutation. OR, odds ratio; aOR, adjusted odds ratio; CI, confidence interval. Values in bold are statistically significant.

### Host blood gene expression associated with different *rpoB* mutations

#### Differentially expressed genes between patients with different *rpoB* mutations

To assess whether different *rpoB* mutations in Mtb isolates are associated with host gene expression, transcriptomic analysis was performed for blood samples from six patients with Mtb harboring H445Y and six age-gender-matched patients with Mtb harboring S450L. Initially, individual sample diversity, which may act as a confounder for the target effects, was estimated using principal component analysis (PCA) of the expression levels of whole genes in the whole blood (Supplementary Figure S1A). The distribution of samples on these two dimensions are shown in Supplementary Figure S1B. Hierarchical clustering further performed on PCA revealed three major clusters (Supplementary Figures S1B,C). A heatmap of log-CPM values for the top 100 genes showing the largest variance indicated the same three clusters (Supplementary Figure S2).

To characterize the gene expression diversity of the blood samples, which may be a confounder in the further analysis, the proportions of 22 blood cell types were estimated by CIBERSORTx, as shown in Supplementary Figure S3. The scores of neutrophils and activated memory CD4 T cells in the absolute mode were highest in cluster 3, followed by clusters 1 and 2 (Supplementary Table S7). No difference in cell type was observed between the H445Y and S450L groups (data not shown). Neutrophils directly measured in the whole blood were also predominant in cluster 3 over clusters 1 and 2 [median = 10.1 G/L (IQR 9.0–10.8) vs. 5.9 (5.5–6.6) vs. 5.8 (5.6–6.3), *p* = 0.0431; Supplementary Table S7].

**Figure 1:**
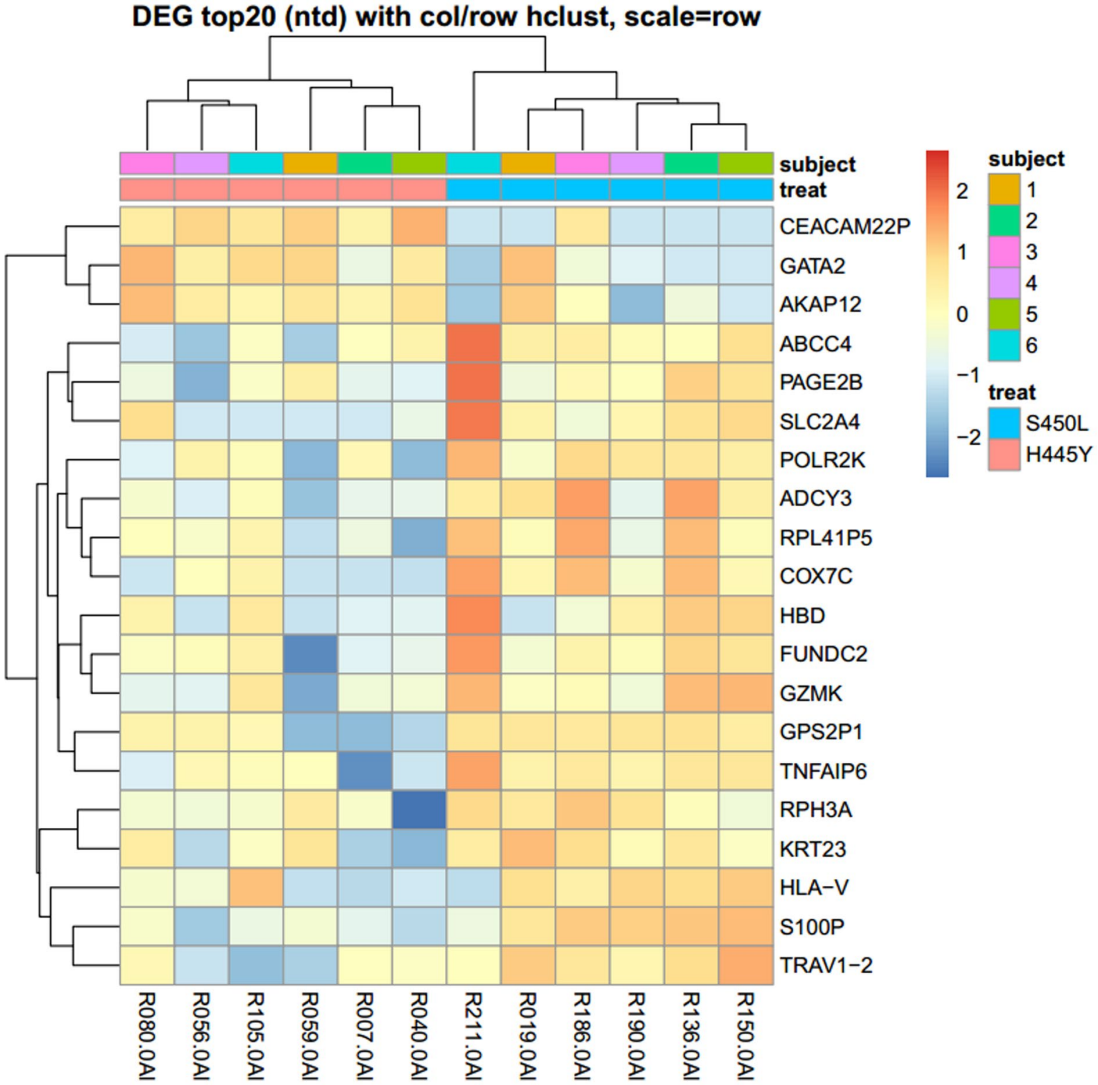
Heatmap of log-CPM values for the top 20 genes differentially expressed between the H445Y and S450L groups. Expression across each gene (each row) has been scaled so that the mean expression is zero. Samples with relatively high expression of a given gene are marked in red; samples with relatively low expression are marked in blue. DEG, Differentially expressed gene.

We initially compared the expression profile of genes in patients infected with Mtb harboring the H445Y mutation with that of the S450L group, without considering aforementioned individual diversity. A heatmap of log-CPM values and read counts in bar graphs for the top 20 DEGs were constructed (Figure 1; Supplementary Figure S4, respectively). Supplementary Table S8 shows the detailed list of DEGs: *S100P*, *FUNDC2*, and *TRAV1-2* were the top three DEGs ranked with *p* values (adjusted *p* values = 0.0022, 0.0022, and 0.0364; log2FC = −2.52, −1.91, and − 1.98, respectively). After adjustment for the PCA-based clusters 1, 2, and 3 as a possible confounding factor, using generalized linear models in the DESeq2 analysis, 50 genes showed differences, of which 33 genes were downregulated in H445Y or upregulated in S450L. Of these, *S100P* was the only gene that had an adjusted *p* value of <0.05 (adjusted *p* values = 0.0396; log2FC = −2.91), followed by *TRAV1-2*, with adjusted *p* value of <0.1 despite the individual diversities.

Pathway enrichment analysis using the Kyoto Encyclopedia of Genes and Genomes system did not show significant results to characterize the above DEGs, whereas GO analysis to identify biological processes indicated that the downregulated genes in the H445Y group were constituent genes of pyrimidine metabolism and/or antiviral response pathways, many of which are also known as IFN-stimulated genes (Supplementary Figure S5; Supplementary Table S9).

#### Differences in genome-wide expression profiles between different *rpoB* mutations

**Figure 2:**
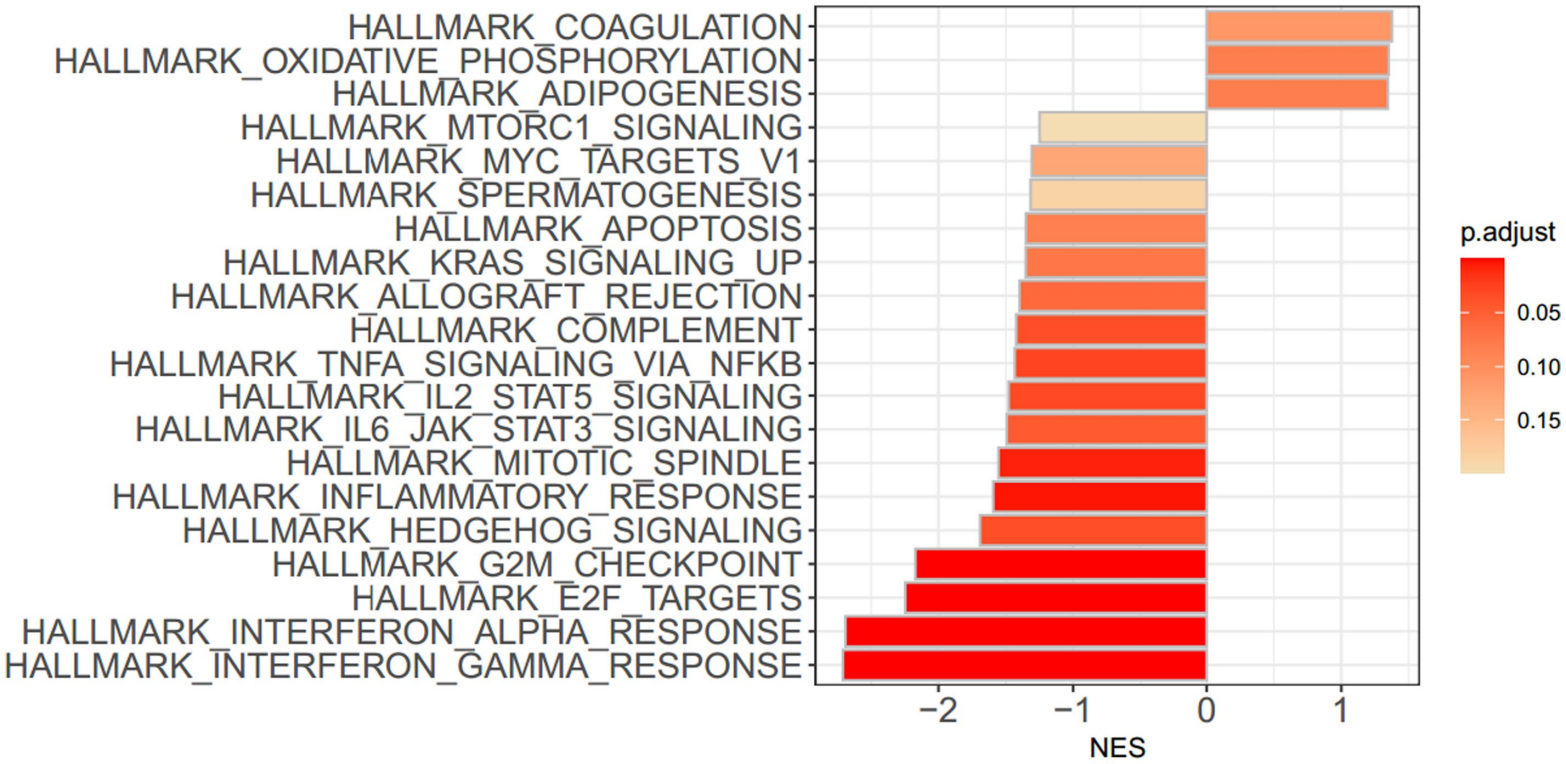
Bar plot showing the 20 most significantly enriched gene sets after adjustment for clustering, obtained by gene set enrichment analysis. Colored bars represent adjusted *p* values. NES, normalized enrichment score.

**Figure 3:**
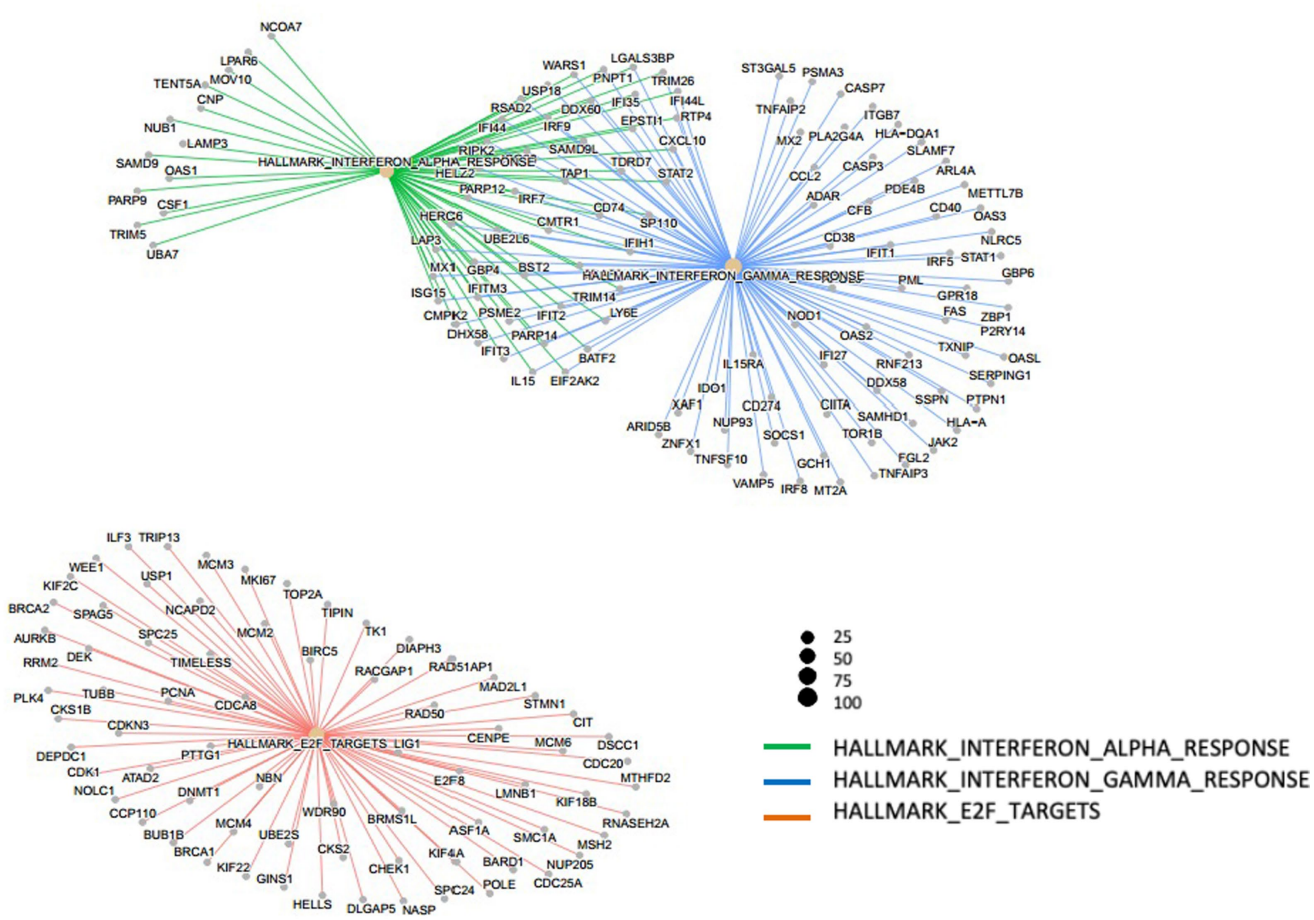
Networking of three out of five hallmark gene sets with strongest significant adjusted *p* values obtained by gene set enrichment analysis after adjustment for clustering.

To identify gene sets showing differences in expression between the H445Y and S450L groups, GSEA was performed on the whole set of expressed genes ranked by their differential expression. GSEA showed that two hallmark gene sets, “hallmark_interferon_gamma_response” and “hallmark_interferon_alpha_response,” had the lowest normalized enrichment scores with significant *p* values in H445Y compared with S450L after the adjustment for the PCA-based clusters (Figure 2; Supplementary Table S10) and before the adjustment (data not shown). Figure 3 shows the networking between these gene sets. Enrichment plots of the top two gene sets are shown in Supplementary Figure S6. By leading edge analysis, the proportion of core genes that accounted for the enrichment signals of these two gene sets were 107/200 (53.5%) and 60/97 (61.9%), respectively, of which 47 genes were shared by the two sets (Supplementary Table S10).

#### Differences in the individual gene expression levels between different *rpoB* mutations

Because the GSEA indicated that the expression levels of IFN-α and-γ response gene sets were the lowest in H445Y compared with S450L, and because elevated expression levels of IFN response genes are associated with active TB disease (Singhania et al., 2018), six IFN-responsive genes, *BATF2*, *SERPING1*, *UBE2L6*, *VAMP5*, *IFI44*, and *STAT1* in the hallmark gene sets (Supplementary Table S10), reported to be associated with TB (Gong et al., 2021), were chosen for validation using qRT-PCR of individual blood samples. *S100P* showed the best *p* value, as the DEG, was also included for the measurements. Among these, the expression levels of *BATF2* and *SERPING1* were significantly lower in the H445Y group than in the S450L group [medium and IQR = 0.48 (0.32–0.58) and 0.73 (0.39–0.82) for *BATF2*, *p* = 0.0328; and 0.60 (0.43–0.86) and 1.14 (0.71–1.70) for *SERPING1*, *p* = 0.0378; Supplementary Figure S7]. The expression levels of the rest of the genes also tended to be lower in the H445Y group compared with the S450L group, although the differences did not reach the significance levels (Supplementary Figure S7).

Using linear regression models after adjustment for gender, age, BMI, disease severity, Mtb lineage/sublineage, and *katG* S315T mutation, H445Y remained inversely associated with *BATF2* and *SERPING1* expression levels (log-transformed values) when compared directly to S450L [coefficient = −0.58 (95% CI −1.05 to −0.10) and − 0.57 (−1.14 to −0.005), respectively]. When they were compared with other *rpoB* mutations, *BATF2*, *SERPING1*, and *VAMP5* showed inversed associations with H445Y (regression coefficients; −0.60 [−0.98 to −0.22], −0.43 [−0.85 to −0.0009], and − 0.35 [−0.69 to −0.009], respectively; Table 5). When TB score ($\sqrt[{4}]{\text{UBE2L6}*\text{BATF2}*\text{SERPING1}*\text{VAMP5}}$) indicating active TB disease (Gong et al., 2021) was calculated based on the expression levels of these four genes using the equation, H445Y was inversely associated with the log value of this score, when compared directly with S450L [coefficient = −0.44 (−0.83 to −0.04)] or with the “other mutation” group [−0.39 (−0.70 to −0.08)]. No significant association was observed between H445Y and *UBE2L6*, *IFI44*, *STAT1*, or *S100P* expression levels (Table 5).

**Table 5:** Association between interferon-inducible gene expression in the blood and *rpoB* mutations in multivariate analysis using regression models.

	Regression coefficient* (95% confidence interval)							
	*BATF2*	*SERPING1*	*VAMP5*	*UBE2L6*	*IFI44*	*STAT1*	*S100P*	TBScore**
Model 1								
H445Y vs. S450L	**−0.58 (−1.05 to − 0.10)**	**−0.57 (−1.14 to − 0.005)**	−0.36 (−0.77 to 0.05)	−0.24 (−0.53 to 0.05)	−0.47 (−1.26 to 0.32)	−0.26 (−0.54 to 0.01)	−0.54 (−1.41 to 0.34)	**−0.44 (−0.83 to − 0.04)**
Model 2								
H445Y vs. other *rpoB* mutations***	**−0.60 (−0.98 to − 0.22)**	**−0.43 (−0.85 to − 0.0009)**	**−0.35 (−0.69 to − 0.009)**	−0.20 (−0.44 to 0.04)	−0.14 (−0.79 to 0.51)	−0.20 (−0.42 to 0.03)	−0.19 (−1.08 to 0.70)	**−0.39 (−0.70 to − 0.08)**
S450L vs. other *rpoB* mutations***	−0.08 (−0.39 to 0.24)	0.08 (−0.27 to 0.43)	−0.001 (−0.28 to 0.28)	−0.0004 (−0.20 to 0.20)	0.11 (−0.43 to 0.65)	0.01 (−0.17 to 0.20)	0.30 (−0.43 to 1.04)	−0.0003 (−0.26 to 0.26)
Model 3								
H445Y vs. no *rpoB* mutation	**−0.42 (−0.80 to − 0.03)**	−0.30 (−0.71 to 0.11)	−0.26 (−0.61 to 0.09)	−0.06 (−0.30 to 0.19)	0.01 (−0.58 to 0.60)	−0.10 (−0.37 to 0.16)	−0.37 (−1.13 to 0.39)	−0.26 (−0.58 to 0.06)
S450L vs. no *rpoB* mutation	0.04 (−0.25 to 0.33)	0.12 (−0.19 to 0.42)	0.04 (−0.22 to 0.30)	0.10 (−0.08 to 0.28)	0.30 (−0.14 to 0.73)	0.12 (−0.07 to 0.32)	0.04 (−0.52 to 0.61)	0.07 (−0.16 to 0.31)
other *rpoB* mutations vs. no *rpoB* mutation	0.18 (−0.08 to 0.44)	0.12 (−0.16 to 0.40)	0.10 (−0.14 to 0.34)	0.13 (−0.03 to 0.30)	0.20 (−0.20 to 0.60)	0.11 (−0.07 to 0.29)	−0.20 (−0.71 to 0.32)	0.13 (−0.08 to 0.35)

*Adjusted for age, gender, body mass index, *katG*-S315T, severity defined by infiltrates presented in the lung zones on chest X-ray, and Mtb lineage/sublineage. **TB score was calculated following Gong et al. (2021). ***Other *rpoB* mutations indicate those other than H445Y and S450L. Mtb: *Mycobacterium tuberculosis*. Values in bold are statistically significant.

### Epidemiological findings associated with interferon-inducible genes in the blood

#### Associations with genotypic clusters and multiple treatment episodes

Low expression levels of *BATF2* and *SERPING1* were associated with the genotypic clusters defined by <6 pairwise SNV difference, both in univariate analysis [OR (95% CI) = 0.44 (0.22–0.88) for *BATF2* and 0.43 (0.22–0.84) for *SERPING1*] and multivariate analysis [adjusted OR (95% CI) = 0.38 (0.16–0.87) for *BATF2* and 0.34 (0.15–0.80) for *SERPING1*], after adjustment for confounding factors (Supplementary Table S11A). Low expression of *BATF2* mRNA was also associated with multiple past treatment episodes according to the logistic regression models [adjusted OR = 0.49 (0.25–0.96)] after adjustment for confounding factors (Supplementary Table S11B).

#### Associations with disease severity

All patients were divided into two groups of disease severity, categorized by the distribution of infiltrates on chest X-ray: mild disease, with lesions limited to <3/6 lung zones, and severe disease with lesions occupying ≥3/6 zones. In the severe group, the expression levels of *BATF2*, *SERPING1*, *VAMP5*, *UBE2L6*, and *STAT1* were significantly lower in the H445Y group compared with the S450L group [median and IQR = 0.32 (0.24–0.35) vs. 0.80 (0.70–0.97) for *BATF2*, *p* = 0.0046; 0.47 (0.34–0.65) vs. 1.17 (1.03–1.81) for *SERPING1*, *p* = 0.0127; 0.58 (0.50–0.69) vs. 0.88 (0.73–1.14) for *VAMP5*, *p* = 0.0127; 0.83 (0.69–1.00) vs. 1.28 (0.98–1.50) for *UBE2L6*, *p* = 0.0127; and 1.33 (1.07–2.01) vs. 2.48 (2.27–2.87) for *STAT1*, *p* = 0.0127, respectively; Supplementary Figure S8]. In the mild disease group, such significant differences were not observed in all markers tested (Supplementary Figure S8). Other indicators of severity, cavity, smear grade, or culture grade were not significantly different between the two mutation groups (data not shown).

## Discussion

In our cohort of sputum smear-positive pulmonary TB patients with history of TB treatment, *rpoB* mutations, particularly *rpoB* S450L and *rpoB* H445Y, were accumulated with high frequencies. *rpoB* H445Y was associated with the ancient Beijing genotype, being clustered and more frequently observed in patients who had received more than one TB treatment episode, whereas S450L and other *rpoB* mutations were not. H445Y was also associated with Rv1830 mutations. By transcriptome analysis of the whole blood from patients before starting retreatment, gene sets involved in the IFN-γ and IFN-α pathways were significantly downregulated in H445Y compared with S450L. The qRT-PCR analysis of the whole blood sample set confirmed that H445Y was inversely associated with IFN-inducible genes, *BATF2* and *SERPING1*, as compared with S450L, even after adjustment for Mtb lineages/sublineages and other possible confounding factors. These findings represented a clinical extension of a recent observation in mouse models, which showed that Mtb strains harboring RIF resistance–conferring mutations modulate host defense systems (Howard et al., 2018).

In our study, a majority of *rpoB* mutations were identified at codons 450 and 445, similar to those reported worldwide (Zaw et al., 2018) and in Vietnam (Caws et al., 2006; Shah et al., 2009; Nguyen et al., 2017; Hang et al., 2019), whereas their distribution differed depending on the Mtb genetic background: modern Beijing strains harbored H445Y much less frequently than S450L, and ancient Beijing strains had H445Y and S450L equally. These findings were consistent with another study that reported extensively drug-resistant TB in China (Zhao et al., 2015).

Among the two major sublineages of Beijing genotype strains, the modern Beijing genotype is predominant in East Asia, widespread in other regions, and frequently associated with genotypic clusters (Emane et al., 2021), indicating recent spread, possibly reflecting higher transmission capability and/or host adaptation (Walker et al., 2013), whereas the ancient Beijing sublineage is limited to smaller regions or countries such as Japan (Seto et al., 2015), Korea (Kang et al., 2010), or Vietnam (Maeda et al., 2020). In our current cohort of TB patients with treatment history in the northern area of Vietnam, the ancient Beijing sublineage, particularly L2.2.AA.3.1, was prevalent and was significantly associated with genetic clusters defined by <12 SNVs, but not <6 SNVs, presumably indicating that this sublineage has spread widely for a long time in the study area, rather than very recently. This tendency was observed even in our previous study on new TB patients in Hanoi (Hang et al., 2019; Maeda et al., 2020).

The H445Y mutation was an independent risk factor for genotypic clusters defined by <6 SNVs and more than one TB treatment episode in our study, indicating patients infected with Mtb strains carrying this mutation are not easily cured and tend to transmit to others. Although the low BMI observed in patients carrying Mtb with the H445Y mutation might be explained by exhaustion of the host caused by repeated TB episodes, the cause-effect relationship is not clear from this study. The success of RIF-resistant strains seems to depend upon the level of resistance, the initial fitness cost, the propensity to accumulate compensatory mutations to alleviate the fitness costs, and recently proposed mutations to regulate recovery from bacterial damage caused by drug exposure, designated as “antibiotic resilience” (Liu et al., 2022). In our study, Mtb strains harboring S450L had a high frequency of compensatory mutations in *rpoA*, *rpoB* or *rpoC*, consistent with another report (Emane et al., 2021), although compensatory mutations do not increase the risk of MDR transmission according to recent reports (Liu et al., 2018; Chen et al., 2022). H445Y-carrying strains were not associated with those compensatory mutations in our study, while coexistent nonsynonymous mutations in Rv1830, also named *resR* or *mcdR*, may promote “antibiotic resilience” and result in the prolonged survival of transmissible bacteria under stress conditions including antibiotic treatment (Liu et al., 2022; Zhou et al., 2022). Indeed, Liu et al. (2022) reported that poor treatment outcome was associated with Rv1830 mutations. In addition, the independent association between Rv1830 mutations and H445Y may imply a possible epistatic interaction, which could play a role in the emergence and spread of drug-resistant Mtb (Borrell and Gagneux, 2011). A future study design to assess the treatment response to Rv1830 mutations and the contribution of epistatic mutations to competitive fitness could shed light on their impact on patient prognosis and drug resistance TB.

Before analyzing blood mRNA signatures in TB patients, we considered individual diversities. PCA of whole gene expression patterns exhibited three hierarchical clusters. In addition, the proportions of neutrophil population estimated by CIBERSORTx and actually counted values in the whole blood were both predominant in cluster 3 over clusters 1 and 2. Although predominance of the neutrophil-driven IFN-inducible gene profile is known in tuberculosis (Berry et al., 2010), there was no difference in this cell type population between the H445Y and S450L groups in our cohort.

Total blood RNA sequencing and downward analyzes showed that *S100P* was the most promising DEG between the H445Y and S450L groups in any conditions tested, but did not reach significance levels by qRT-PCR expression analysis of the whole sample set. Nevertheless, *S100P* was also observed in the TB-related whole blood mRNA signature in another study (Verhagen et al., 2013). S100P is a member of S100 protein family and functions as a regulator of diverse cellular processes, especially tumor cell lines and tissues. Transcriptional regulation of *S100P* is complicated (Gibadulinova et al., 2011). *TRAV1-2* was another DEG candidate between the H445Y and S450L groups despite individual diversities, although adjusted *p* values below 0.1 and T cell receptor (TCR) repertoires were not analyzed in this study. TRAV1-2 is used as a set of TRAV1-2/TRAJ12/20/33 (Napier et al., 2015), comprising the invariant TCRα chain of mucosal-associated invariant T cells. Notably, recent TCR sequencing analysis revealed that *TRAV1-2* was part of mycobacteria-reactive TCRα, and its expression was associated with TB disease progression or control (Musvosvi et al., 2023).

GSEA after adjusting for PCA-based clusters revealed that two gene sets involved in the type I and II IFN pathways were significantly enriched not in H445Y but in S450L, irrespective of individual diversities, while the majority of these genes are activated in both types of IFN signaling pathway. GO enrichment analysis also indirectly supported the contribution of IFN pathways. Predominance of type I IFN-inducible blood transcriptional signatures correlates with TB disease severity and progression (Moreira-Teixeira et al., 2018; Singhania et al., 2018), and type II IFN (IFN-γ) is an important immunomodulator in the pathogenesis of TB (Chin et al., 2017). Thus IFN-inducible genes have been considered as promising blood-based biomarkers for TB (Singhania et al., 2018). The four genes, *BATF2*, *SERPING1*, *UBE2L6*, and *VAMP5*, which were reported to have high diagnostic value for active TB (Gong et al., 2021), are involved in these two enriched pathways. Of these, *BATF2* has often been nominated as one of the top DEGs in active TB (Gupta et al., 2020; Tabone et al., 2021). In our study, the expression levels of three of these four genes in the whole blood were inversely associated with H445Y’s presence even after adjustment for confounding factors, including Mtb lineages/sublineages, and validated the result obtained from RNA sequencing, suggesting that blood transcriptional signatures are affected by pathogenic variants in a clinical setting.

Howard et al. (2018) demonstrated that the infection of strains carrying *rpoB* S450L or wild-type *rpoB* resulted in exacerbated pulmonary inflammation, but *rpoB* H445Y modulated bacterial cell wall lipids, and thus, based on the reprogramming of macrophage metabolism, infection with Mtb harboring H445Y induced host defense pathways differently from infection with wild-type or S450L strains in murine models. Our study results partly supported their findings because host gene expression profiles were different between H445Y and S450L. Downregulation of IFN-inducible genes in the H445Y group was shown in the subgroup with extensive lung lesions and this negative association was observed even after adjustment for Mtb lineage/sublineage and disease severity. The host immune response can be suppressed depending on Mtb lineages/sublineages, which may contribute to increased virulence of Mtb and more efficient transmission (Coscolla and Gagneux, 2014). A similar mechanism may underlie the H445Y group. Further investigation would be necessary to determine whether low levels of IFN response genes in the H445Y group was a result of exhaustion due to multiple TB episodes or a cause of altered immune response reported in murine models and the subsequent recurrence and transmission to others.

Our study has some limitations. First, the sample numbers for previously treated TB patients in our study were relatively small. However, the catchment area was limited to one city; therefore, we were able to assess local transmission of TB, analyzing genotypic clusters and drug resistance intensively. Second, our clinical and epidemiological findings have not been corroborated by an *in vitro* growth model. It has not been established due to insufficient resources to assure biosafety in our local setting, and was beyond the scope of this study. Third, the treatment history of active TB was obtained from a structured questionnaire. Although healthcare workers had been trained and carefully conducted face-to-face interview and any ambiguous information was reconfirmed by direct contact or further checking registration logbooks in district health centers, the recall bias may not be negligible. Fourth, Mtb isolates from past episodes were not available, and we thus had no information whether the infection represented relapse or reinfection, or exactly when the drug resistance mutations were generated. Nevertheless, to our understanding, this is the first report on the independent association between repeated TB treatment and *rpoB* mutations in a clinical setting.

In conclusion, the distribution of *rpoB* mutations was different depending on the Beijing sublineages isolated from TB patients with treatment history. It is conceivable that epistatic interactions with *rpo*B mutations and alteration of host responses, including downregulation of the IFN response genes, influence multiple treatment episodes or genotypic clusters indicating recent spread, possibly reflecting higher transmission capability and/or host adaptation. These findings strengthen the importance of further research into host-pathogen relationships in drug-resistant TB for better TB control, including the development of new vaccines or therapeutic agents.

## Data availability statement

The data presented in the study are deposited in the DDBJ repository, https://www.ddbj.nig.ac.jp/, accession numbers PRJDB15583 and PRJDB15892.

## Ethics statement

The studies involving human participants were reviewed and approved by the Ethical Committees of the Hanoi Department of Health, Vietnam, the National Center for Global Health and Medicine, and the Research Institute of Tuberculosis, Japan Anti-Tuberculosis Association, Japan (RITIRB29-35). The patients/participants provided their written informed consent to participate in this study.

## Author contributions

NTLH planned and supervised on-site implementation, performed the data analyzes, wrote and finalized the manuscript. MH performed sequencing and real-time RT-PCR, analyzed sequencing results, wrote and finalized the manuscript. SM coordinated and supervised microbiological data collection and quality control. PT, HH, PA, NTH, and VC supervised on-site implementation. NPH supervised pathogen data collection. DT supervised host data collection. NKo read chest X-ray films. KW and AM provided technical support for sequencing. SS provided support for microbiological analyzes. NKe conceptualized the project, wrote in-house scripts for data analyzes, performed the analyzes, corrected and finalized the manuscript. All authors read and agreed to the published version of the manuscript.

## Funding

This research was supported by AMED under Grant Numbers JP22wm0225011 (Global Research Infrastructure, Collaborative Research via Overseas Research Centers) and JP22fk0108128 (Research Program on Emerging and Re-emerging Infectious Diseases), and also supported by JSPS KAKENHI under Grant Number JP20KK0197 [Fostering Joint International Research (B)].

## Conflict of interest

The authors declare that the research was conducted in the absence of any commercial or financial relationships that could be construed as a potential conflict of interest.

## Publisher’s note

All claims expressed in this article are solely those of the authors and do not necessarily represent those of their affiliated organizations, or those of the publisher, the editors and the reviewers. Any product that may be evaluated in this article, or claim that may be made by its manufacturer, is not guaranteed or endorsed by the publisher.

## Footnotes

^1^ https://www.who.int/publications/i/item/9789240061729

^2^ https://github.com/lh3/bwa

^3^ https://github.com/broadinstitute/gatk/releases

^4^ https://github.com/jodyphelan/TBProfiler

^5^ https://www.gencodegenes.org/human/

^6^ https://github.com/alexdobin/STAR

^7^ https://cibersortx.stanford.edu

^8^ https://github.com/talgalili/gplots

^9^ https://www.who.int/publications/i/item/9789240028173
