# Supplementary material for: Host-pathogen relationship in retreated tuberculosis with major rifampicin resistance–conferring mutations
Source: Front Microbiol. 2023 Jul 4;14:1187390. doi: 10.3389/fmicb.2023.1187390 (PMC10352910; doi:10.3389/fmicb.2023.1187390)
Supplement: Supplementary file 2 [file Presentation_1.PPTX]

## Slide 1
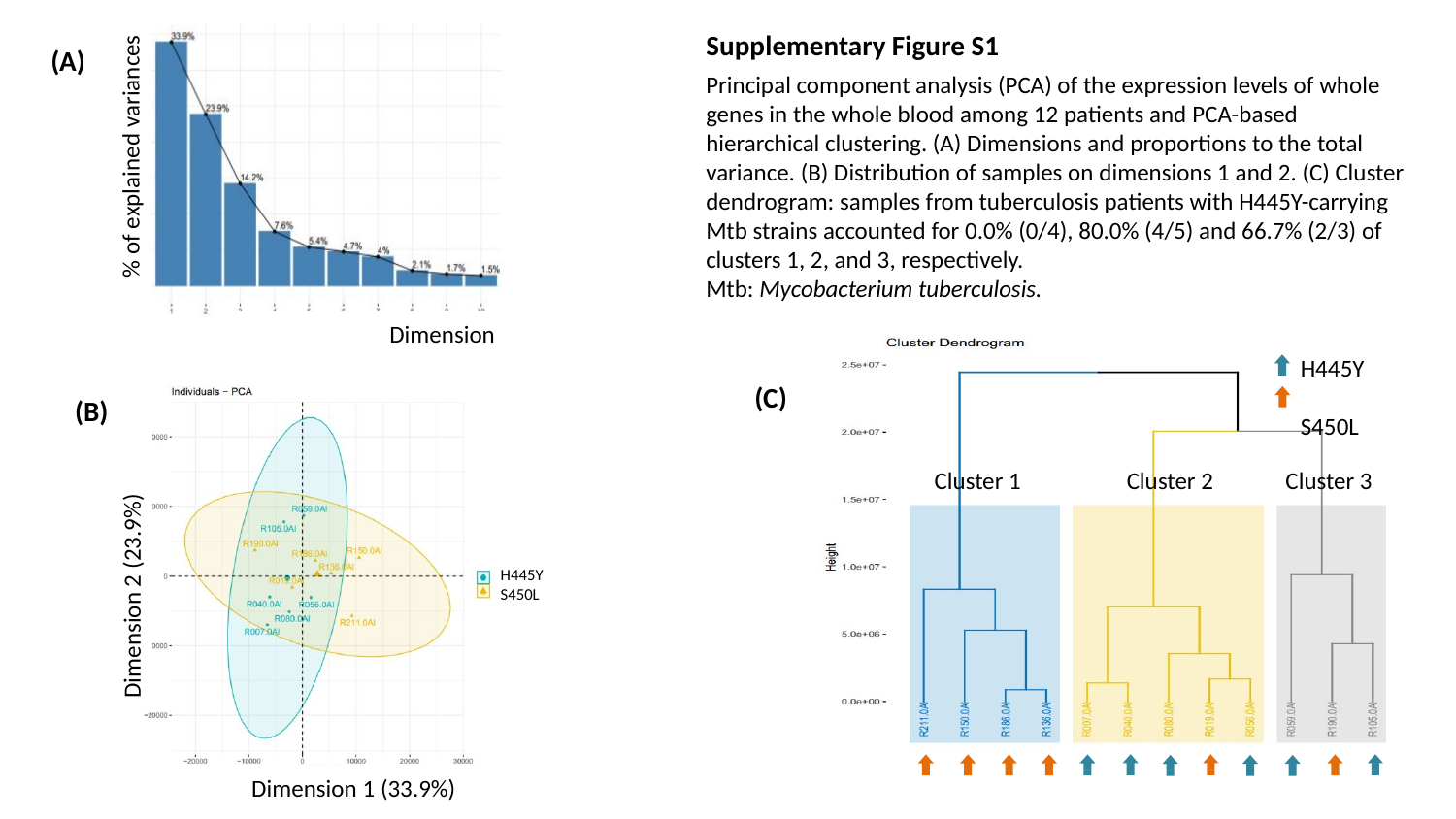

Supplementary Figure S1
% of explained variances
Dimension
(A)
Principal component analysis (PCA) of the expression levels of whole genes in the whole blood among 12 patients and PCA-based hierarchical clustering. (A) Dimensions and proportions to the total variance. (B) Distribution of samples on dimensions 1 and 2. (C) Cluster dendrogram: samples from tuberculosis patients with H445Y-carrying Mtb strains accounted for 0.0% (0/4), 80.0% (4/5) and 66.7% (2/3) of clusters 1, 2, and 3, respectively.
Mtb: Mycobacterium tuberculosis.
H445Y
S450L
(C)
(B)
H445Y
S450L
H445Y
S450L
Dimension 2 (23.9%)
Dimension 1 (33.9%)
 Cluster 1 Cluster 2 Cluster 3

## Slide 2
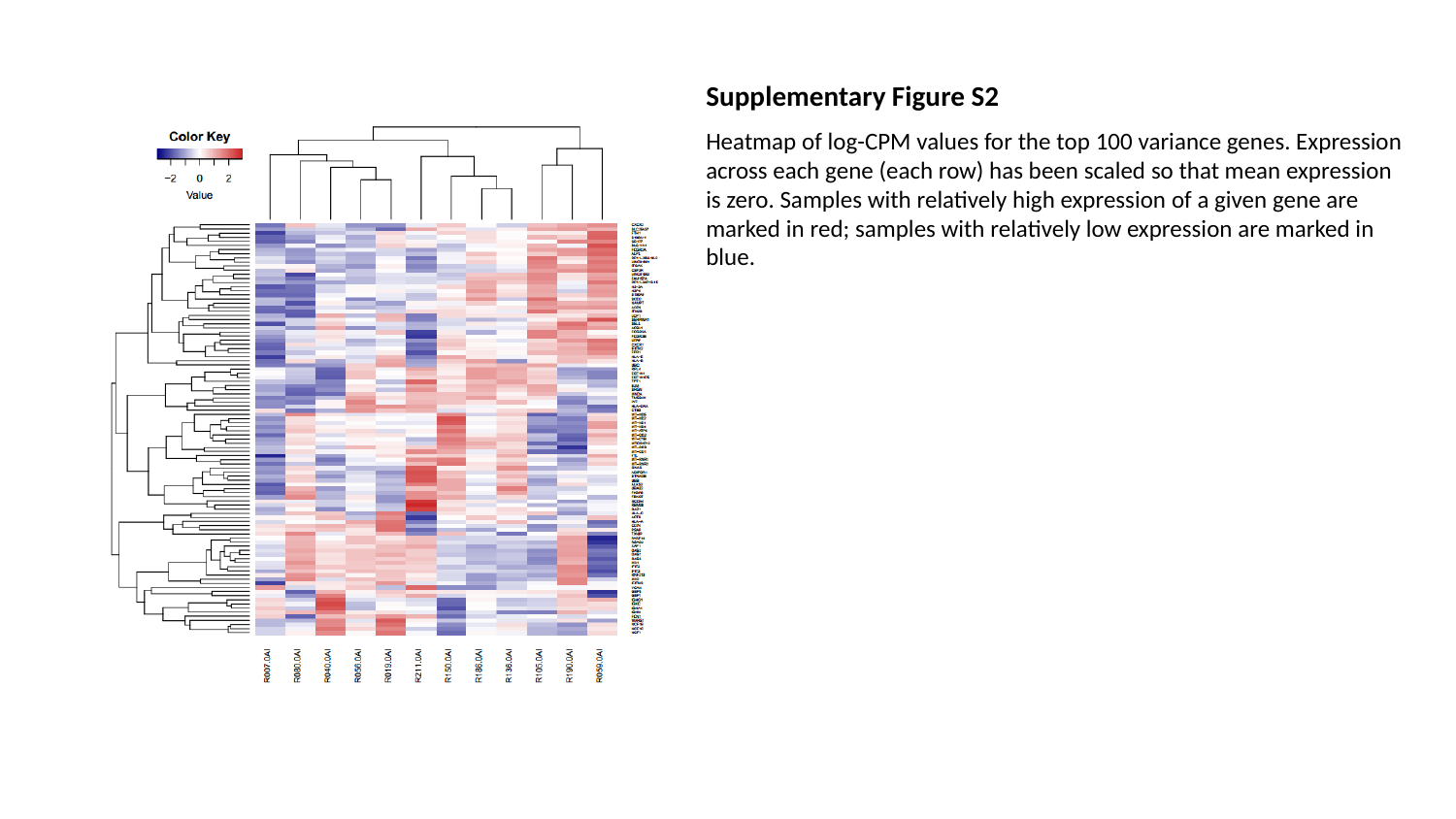

Supplementary Figure S2
Heatmap of log-CPM values for the top 100 variance genes. Expression across each gene (each row) has been scaled so that mean expression is zero. Samples with relatively high expression of a given gene are marked in red; samples with relatively low expression are marked in blue.

## Slide 3
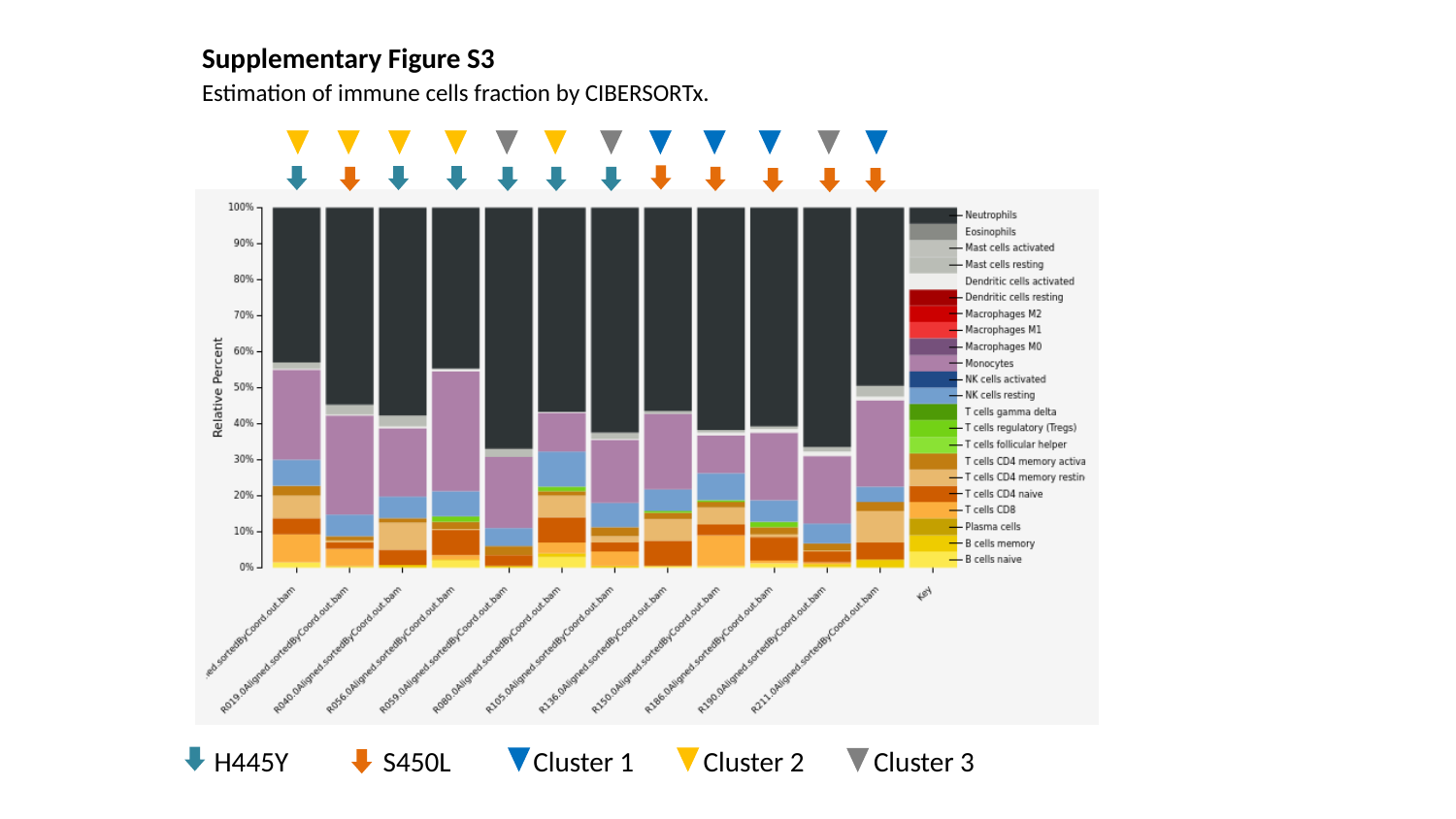

Supplementary Figure S3
Estimation of immune cells fraction by CIBERSORTx.
H445Y S450L
Cluster 1 Cluster 2 Cluster 3

## Slide 4
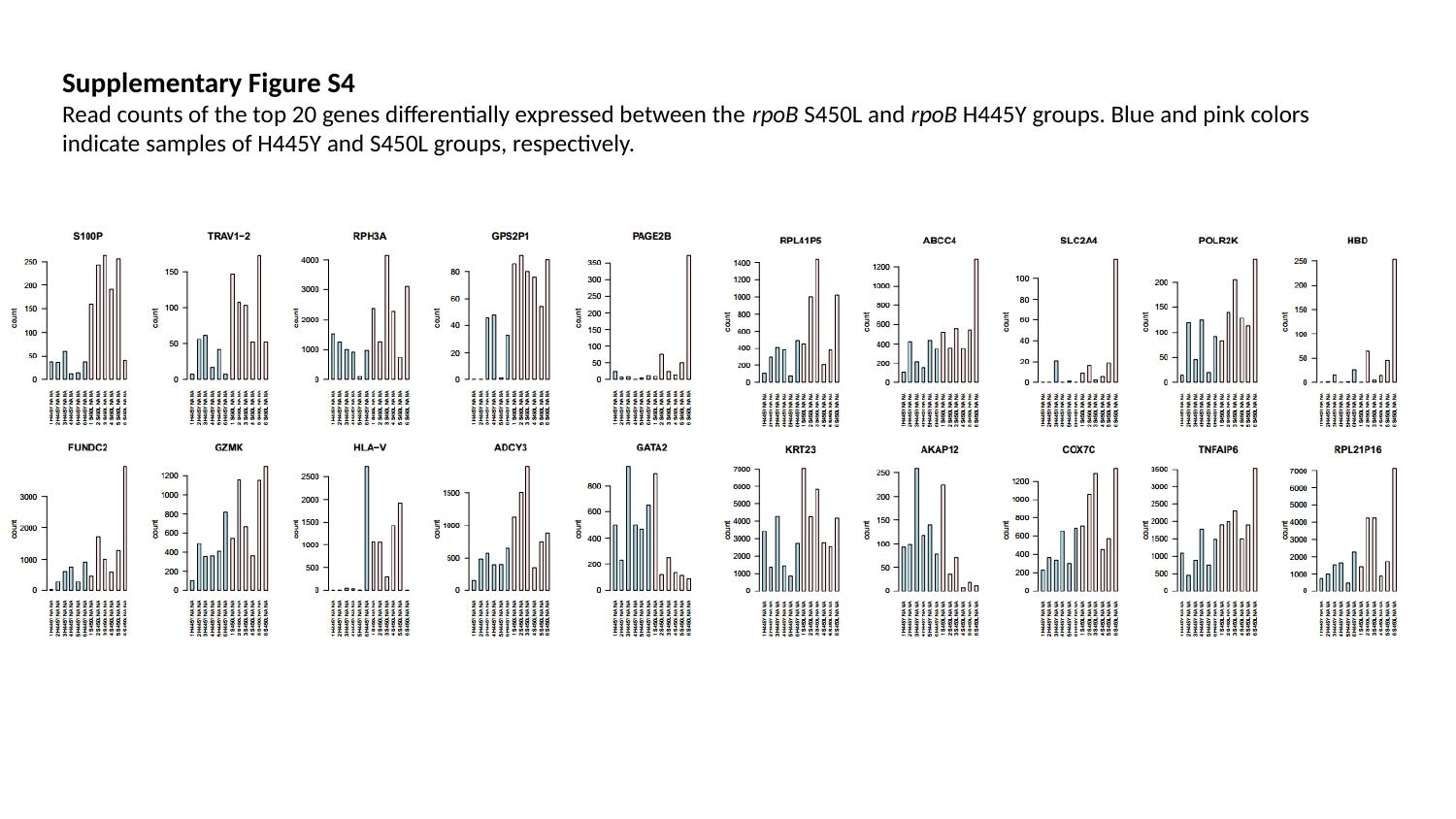

Supplementary Figure S4
Read counts of the top 20 genes differentially expressed between the rpoB S450L and rpoB H445Y groups. Blue and pink colors indicate samples of H445Y and S450L groups, respectively.

## Slide 5
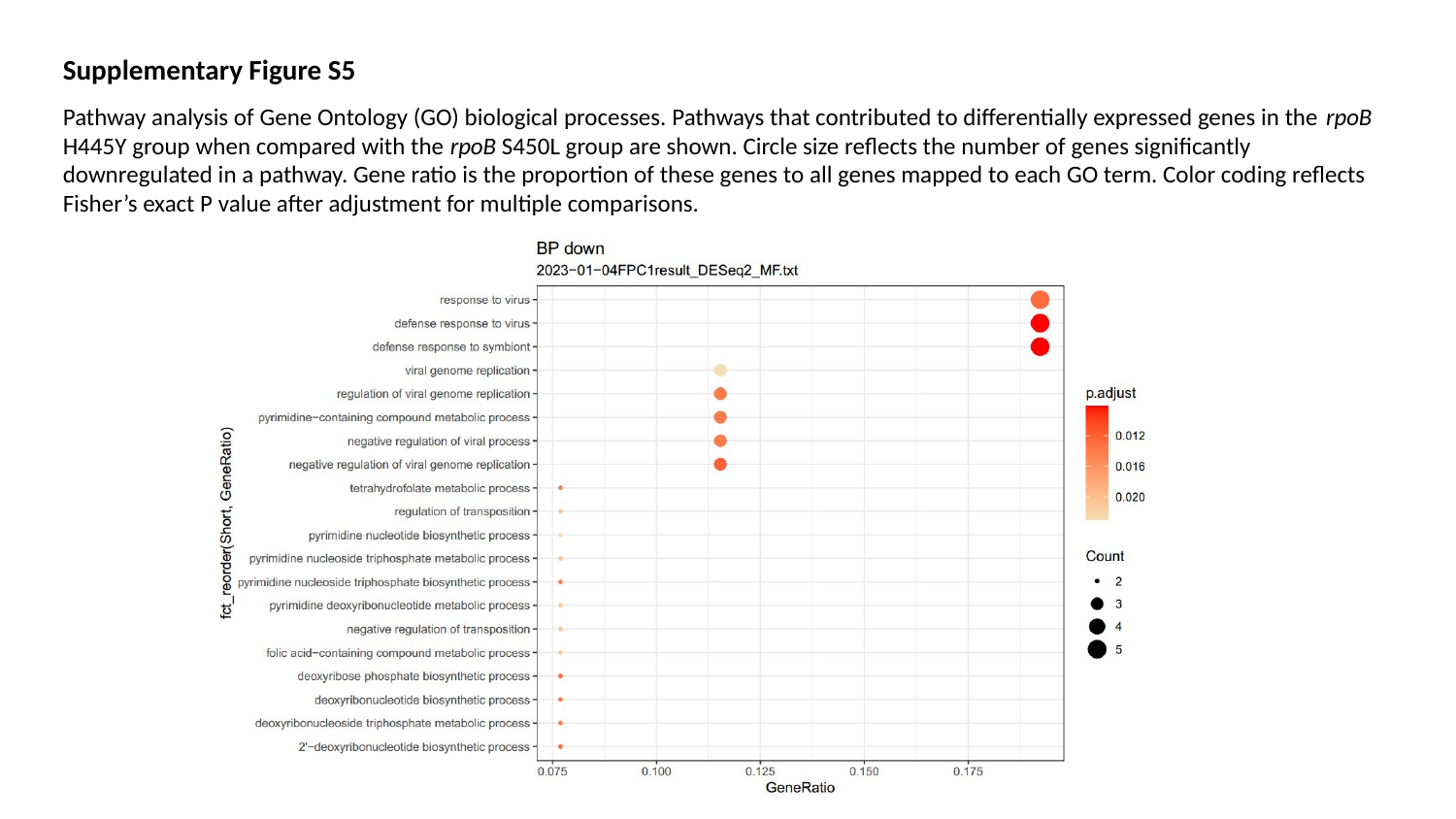

Supplementary Figure S5
Pathway analysis of Gene Ontology (GO) biological processes. Pathways that contributed to differentially expressed genes in the rpoB H445Y group when compared with the rpoB S450L group are shown. Circle size reflects the number of genes significantly downregulated in a pathway. Gene ratio is the proportion of these genes to all genes mapped to each GO term. Color coding reflects Fisher’s exact P value after adjustment for multiple comparisons.

## Slide 6
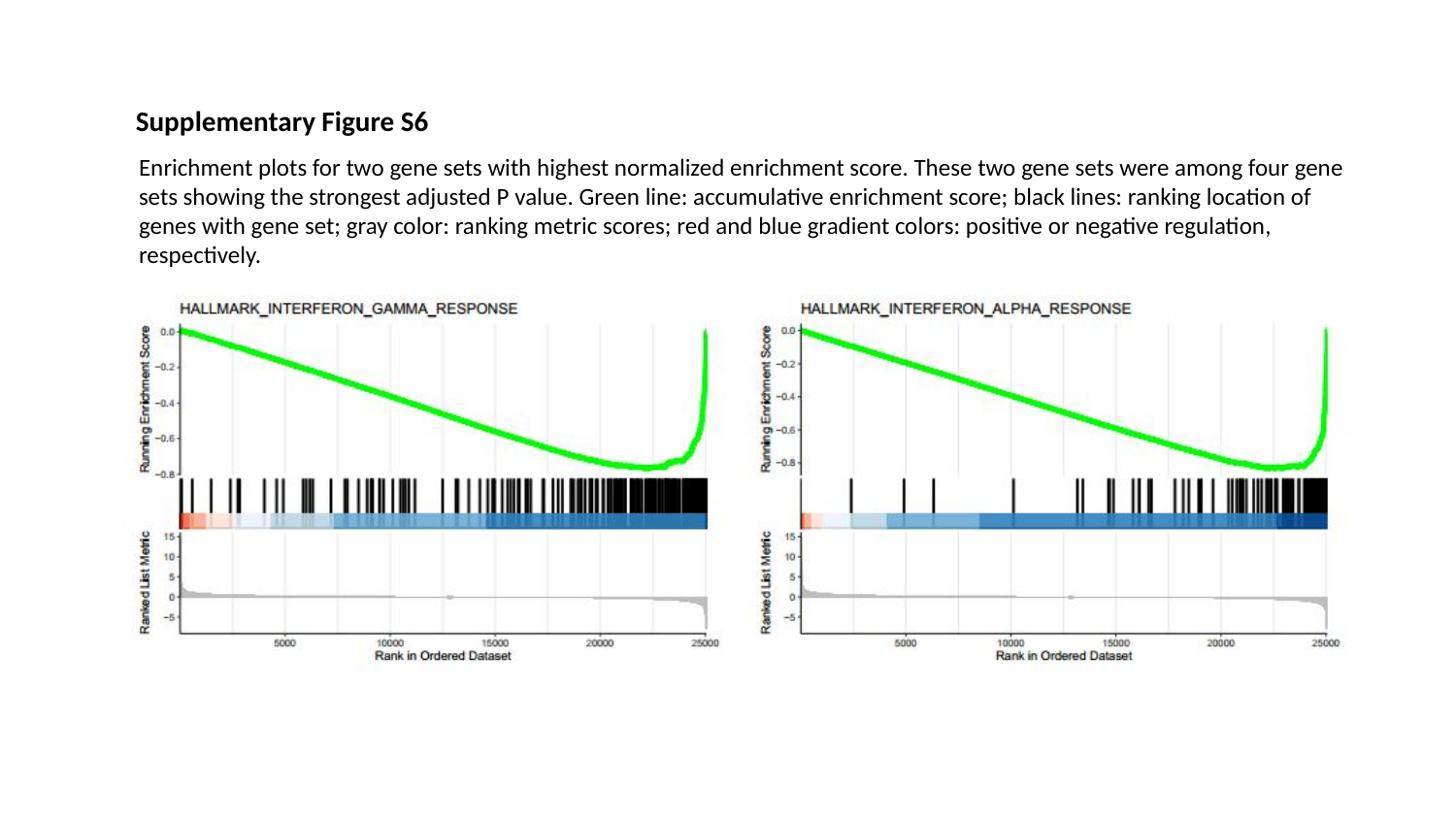

Supplementary Figure S6
Enrichment plots for two gene sets with highest normalized enrichment score. These two gene sets were among four gene sets showing the strongest adjusted P value. Green line: accumulative enrichment score; black lines: ranking location of genes with gene set; gray color: ranking metric scores; red and blue gradient colors: positive or negative regulation, respectively.

## Slide 7
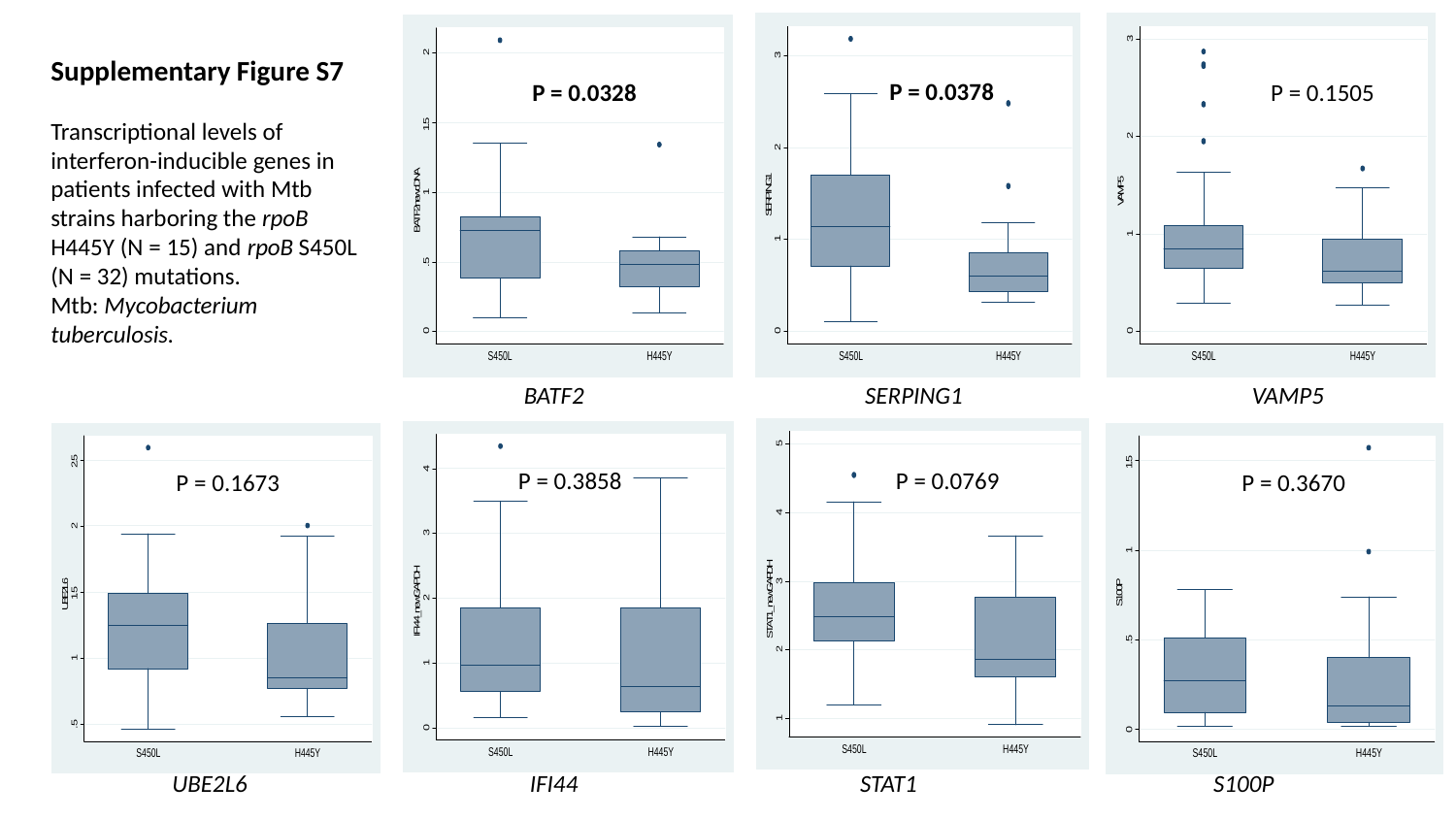

Supplementary Figure S7
Transcriptional levels of interferon-inducible genes in patients infected with Mtb strains harboring the rpoB H445Y (N = 15) and rpoB S450L (N = 32) mutations.
Mtb: Mycobacterium tuberculosis.
P = 0.0378
P = 0.1505
P = 0.0328
BATF2		 SERPING1		VAMP5
P = 0.3858
P = 0.0769
P = 0.1673
P = 0.3670
UBE2L6		 IFI44		 STAT1 		 S100P

## Slide 8
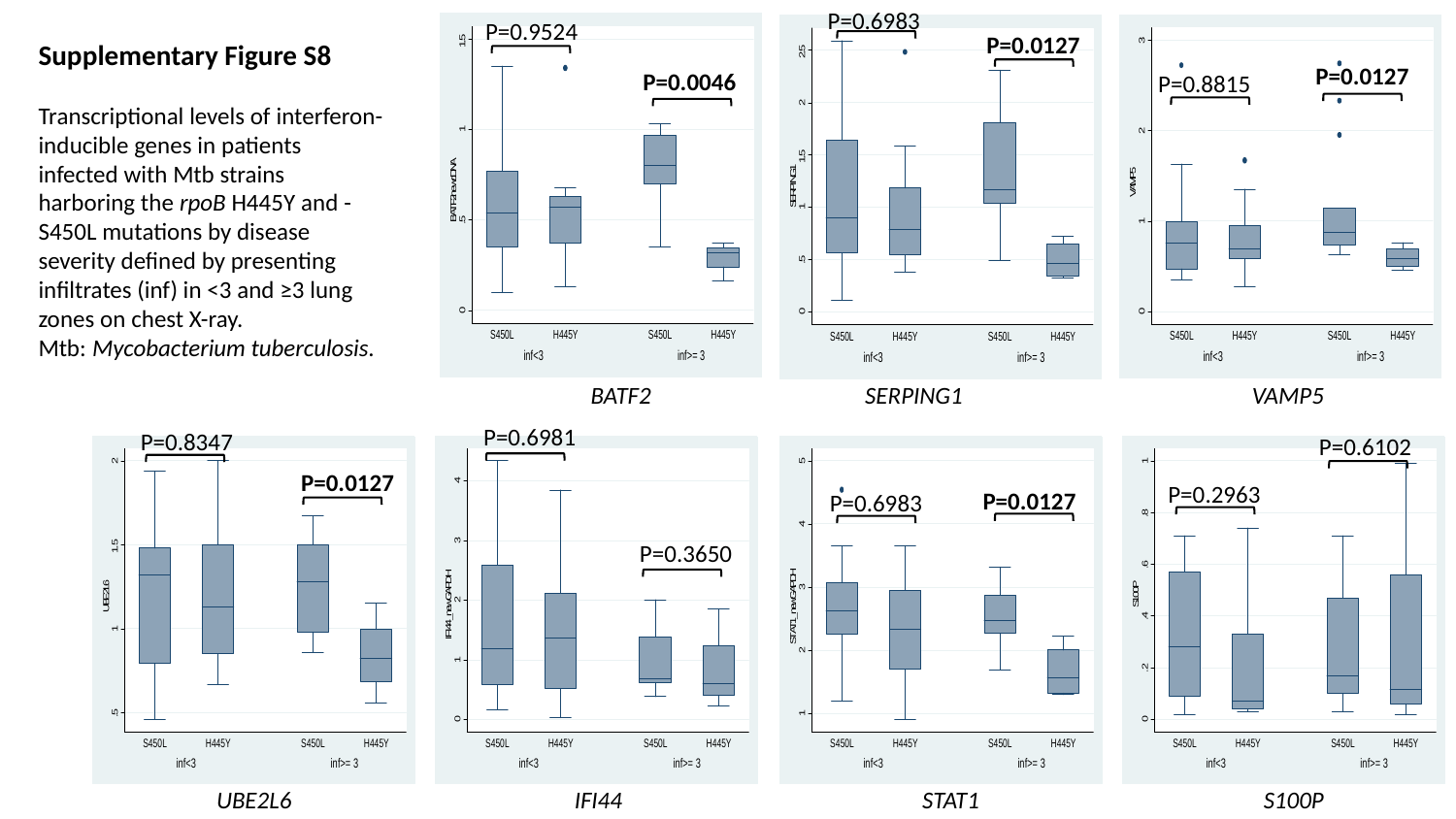

P=0.6983
P=0.9524
P=0.0127
P=0.0127
P=0.0046
P=0.8815
 BATF2		 SERPING1		VAMP5
Supplementary Figure S8
Transcriptional levels of interferon-inducible genes in patients infected with Mtb strains harboring the rpoB H445Y and -S450L mutations by disease severity defined by presenting infiltrates (inf) in <3 and ≥3 lung zones on chest X-ray.
Mtb: Mycobacterium tuberculosis.
P=0.6981
P=0.8347
P=0.6102
P=0.0127
P=0.2963
P=0.0127
P=0.6983
P=0.3650
 UBE2L6		 IFI44		 STAT1 		 S100P
